# Post-acute traumatic brain injury recovery: architecture of an illustrative curated evidence registry

**DOI:** 10.3389/fneur.2026.1979438

**Published:** 2026-10-19

**Authors:** Fangchuan Cao, Liangdie Xu, Weiyang Wang, Ting Yu, Guangda Xu

**Affiliations:** Department of Neurosurgery, Tiantai People’s Hospital of Zhejiang Province (Tiantai Branch of Zhejiang Provincial People’s Hospital), Taizhou, Zhejiang, China

**Keywords:** clinical decision context, evidence registry, implementation, post-acute recovery, rehabilitation, traumatic brain injury

## Abstract

**Introduction:**

Traumatic brain injury (TBI) recovery is shaped by secondary neuroinflammatory and neurovascular responses, injury severity, pre-injury health, rehabilitation timing and care setting. Yet rehabilitation evidence is commonly organized around interventions rather than the clinical decision context in which an intervention is considered.

**Methods:**

We developed TBI-RECOVER to describe the architecture of a curated post-acute TBI recovery evidence registry across clinical decision-context signals, recovery domains, outcomes, care settings and nominal source roles. English-language PubMed records from database inception to 21 August 2026 were retrieved through seven complementary recovery-oriented search strands. Exact queries, PMIDs, XML metadata, retrieval failures and rule-based triage decisions were archived. An illustrative 50-source curated registry of guidelines, consensus documents and high-level evidence syntheses was assembled from returned records. Each entry received a primary recovery domain, a nominal source-role category, severity, recovery-time, care-setting, age and comorbidity reporting signals, and one or more outcome labels. Selection rationales were recorded for every entry; no numerical domain quota was used.

**Results:**

The curated registry comprised 50 sources after title/abstract and multidisciplinary re-audit: 10 guidelines or consensus documents, 16 intervention syntheses, 10 implementation or service-evidence sources, 13 measurement or prognostic-evidence sources and one protocol or methods source. Forty-six entries occupied the score 12 or higher triage stratum. Independent stratified manual relevance validation confirmed the original triage assignments, and multidisciplinary review confirmed the prespecified source-role and primary-domain classifications. The stored score was reproduced exactly for all 2,529 parsed records. Age language was absent in 23 sources, and no molecular/biochemical marker, cell-type or brain-region signal was present in the curated title/abstract metadata.

**Discussion:**

TBI-RECOVER provides a transparent structure for locating high-level recovery evidence, specifying clinical decision-context fields for future synthesis, and designing stratified rehabilitation and implementation studies.

## Introduction

TBI is not a single recovery problem. Primary mechanical injury is followed by metabolic, vascular and innate immune responses that intersect with neuroplasticity, rehabilitation, symptom persistence and social reintegration over multiple timescales (1–3). Current care therefore requires decisions that extend beyond acute stabilization: when to begin rehabilitation; how to match cognitive, physical, behavioural and psychosocial interventions to recovery needs; and how to deliver care across inpatient, outpatient, community, vocational and digital settings. Early rehabilitation guidance underscores the importance of this transition, but the organization and evidentiary maturity of post-acute decisions remain heterogeneous (4).

Recent TBI guidance and syntheses demonstrate substantial activity across individual recovery needs. Contemporary guidance addresses persisting symptoms after mild TBI, cognitive pharmacotherapy, mental health, behaviour, post-traumatic headache, exercise and occupational therapy, while consensus work also addresses service organization and rehabilitation after concussion. These sources are decision anchors, but their populations, outcome definitions, timing and implementation assumptions vary substantially (5–14). Non-drug programmes also vary in dose, intensity, delivery setting and outcome definition, whereas pharmacological strategies are commonly symptom-specific and do not by themselves establish gains in function, participation or long-term recovery. These limitations differ by injury severity and recovery stage, providing the theoretical rationale for a decision-context registry rather than an undifferentiated treatment list.

Focused syntheses similarly exist for fatigue, sleep, persistent symptoms, chronic pain, vestibular rehabilitation, physical activity and exercise; cognitive rehabilitation; mental-health outcomes and psychological interventions; and social, vocational and caregiver outcomes (15–42). However, coverage in a named domain does not establish that its effects are comparable, that outcomes are measured consistently, or that an intervention has been tested in the recovery phase and setting where it would be used. These caveats are particularly important in TBI, where severity labels are imprecise, recovery trajectories vary, and symptom clusters can coexist with social and structural barriers.

A recovery-oriented synthesis must preserve the coordinates of the decision rather than only the name of the intervention. The same symptom can occur in the first weeks after injury or years later; the same cognitive programme can be delivered in an inpatient unit, outpatient clinic, school, workplace or digital platform; and identical numerical changes can have different implications for returning to employment, supporting a caregiver or organizing a rehabilitation service. We therefore use clinical decision context to refer to reported injury severity, recovery timing, functional need, symptom burden and care setting. This wording avoids treating metadata-derived descriptors as validated biological or clinical phenotypes.

Evidence maps and guideline collections have characterized important parts of the field, but a recovery-oriented resource must expose the links among clinical decision context, recovery domain, patient-relevant outcome, care setting and source role. The focused objective of TBI-RECOVER was to describe the architecture of a transparent illustrative curated registry for post-acute TBI recovery, while preserving the retrieval and annotation trail needed to formulate precise clinical, translational and implementation questions.

## Methods

### Study design and analytic boundary

TBI-RECOVER was designed as a source-level evidence-registry study. The unit of retrieval was the PubMed record, and the unit of analysis was a curated high-level source. The central question was: how is post-acute TBI recovery evidence represented across clinical decision-context signals, recovery domains, patient-relevant outcomes, care settings and nominal source roles? This design does not estimate comparative treatment effects or develop a patient-level prognostic model. Table 1 specifies the scope, registry construction and interpretation framework.

**Table 1:** Study scope and registry-construction framework for TBI-RECOVER.

Element	Specification
Study object	Source-level evidence-registry study describing the architecture of an illustrative curated registry for post-acute TBI recovery.
Population and age terminology	Adults and older adolescents aged 17–19 years. Paediatric, adult, mixed-age and unstated age language are retained as explicit metadata categories.
Recovery window	Seven days to five years after injury, specified as an operational boundary spanning early rehabilitation, transition, community recovery and persistent symptoms.
Retrieval base	English-language PubMed-indexed high-level evidence retrieved from database inception through 21 August 2026.
Curated registry	Fifty contemporary sources selected to represent pre-specified recovery decision contexts. No numerical domain quota was used; selection rationales are reported in Supplementary material S15.
Source-role annotation	Guideline or consensus; intervention synthesis; implementation or service evidence; measurement or prognostic evidence; protocol or methods source. These are nominal categories.
Validation scope	Archived retrieval, bibliographic identity verification, exact score recomputation for 2,529 records, independent human relevance validation and multidisciplinary review of all 50 curated sources; prospective patient-level clinical validation was not performed.

### Population and clinical scope

Post-acute recovery was operationally defined as 7 days to 5 years after injury, spanning early rehabilitation, care transition, community recovery and persistent symptoms. The population of interest was adults and older adolescents aged 17–19 years with TBI. Sources with paediatric, adult, mixed-age or unstated age language were retained as explicit reporting categories. The registry included multidisciplinary rehabilitation; cognitive rehabilitation; symptoms, sleep and physical recovery; mental health and behaviour; participation and social recovery; implementation and access; and outcome measurement. Acute neurosurgery, resuscitation fluids, osmotherapy, acute neuroprotection and intensive-care interventions were not counted as post-acute rehabilitation interventions. Their potential relevance as treatment-history context is shown in Figure 1B, rather than being conflated with rehabilitation effectiveness.

**Figure 1:**
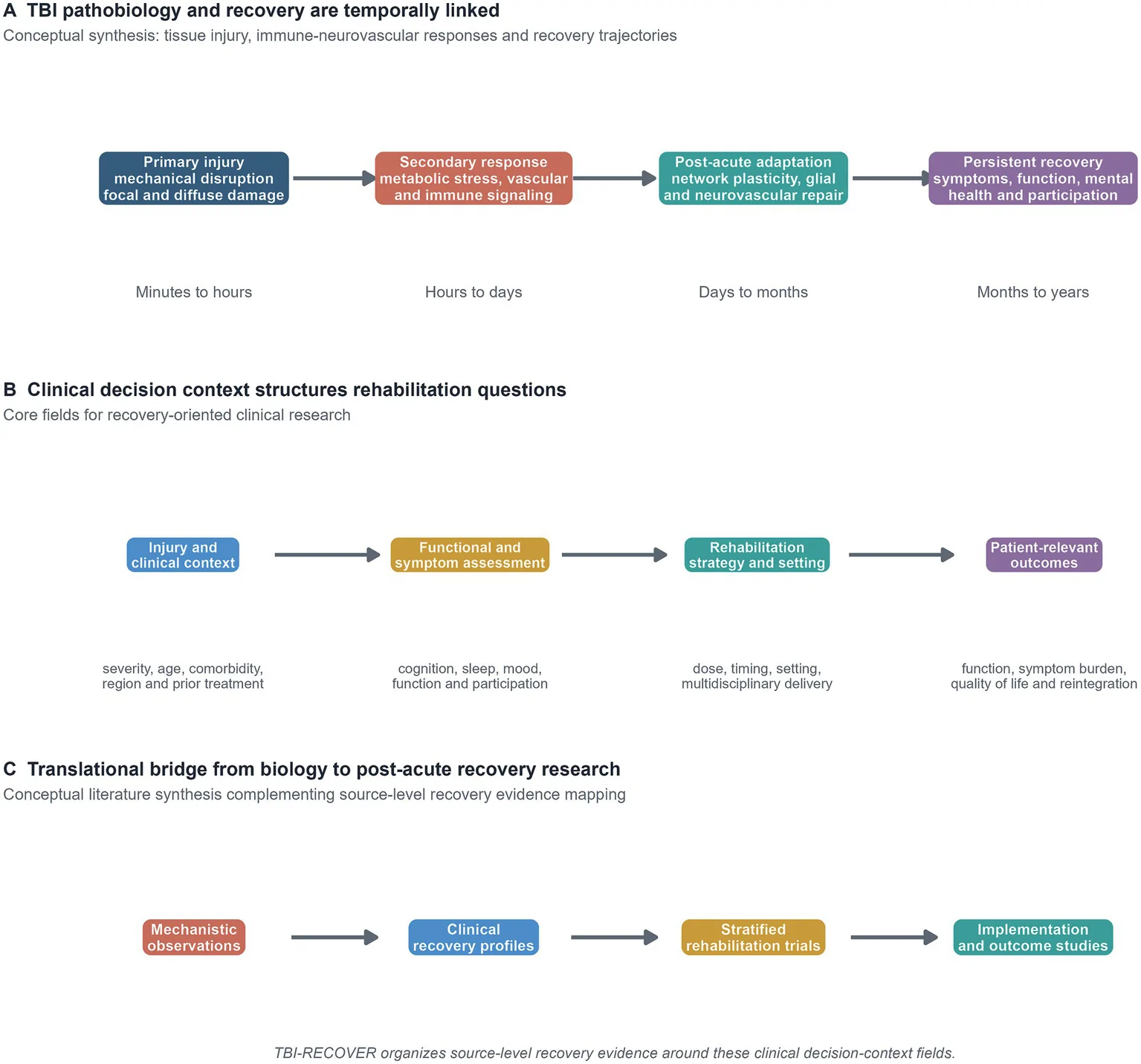
Conceptual framework linking TBI pathobiology, post-acute recovery and translational rehabilitation research. **(A)** Conceptual temporal relation between tissue injury, secondary pathobiology and recovery. **(B)** Clinical decision-context fields that structure rehabilitation questions. **(C)** Translational pathway from mechanistic observations to prospective rehabilitation and implementation research.

### Search strategy

*Search strategy and provenance*: The PubMed search was conducted through the NCBI E-utilities on 21 August 2026. Seven complementary strands covered recovery rehabilitation and function; cognitive rehabilitation; physical symptoms, sleep and vestibular care; mental health and behavioural recovery; participation, vocational and social recovery; care delivery, implementation and access; and outcome measurement or prognosis. Each strand combined a TBI population block with recovery-context or domain terms, an English-language filter and a high-level-evidence filter based on publication type or title-level descriptors. The full query strings, broad retrieval-probe counts, high-level retrieval counts, search date and endpoint are preserved in Supplementary materials S1, S2. Summed strand counts are overlapping retrieval counts rather than independent-study denominators.

### Metadata acquisition and reconciliation

PubMed ESearch supplied per-strand PMIDs, which were deduplicated before batched EFetch retrieval of XML metadata. We parsed title, abstract, journal, publication year, authors, DOI, publication types, Medical Subject Headings and search-strand provenance. The raw record table, PMID-to-strand mapping and record-level field completeness are provided in Supplementary materials S3–S5. We retained records that did not have all metadata fields, and we retained a separate list of PMIDs for which the EFetch endpoint did not return parseable XML (Supplementary material S7). This decision prevents silent loss of records and allows future reruns to reconcile changes in PubMed indexing. The retrieval flow is reported in Figure 2A and Supplementary material S8.

**Figure 2:**
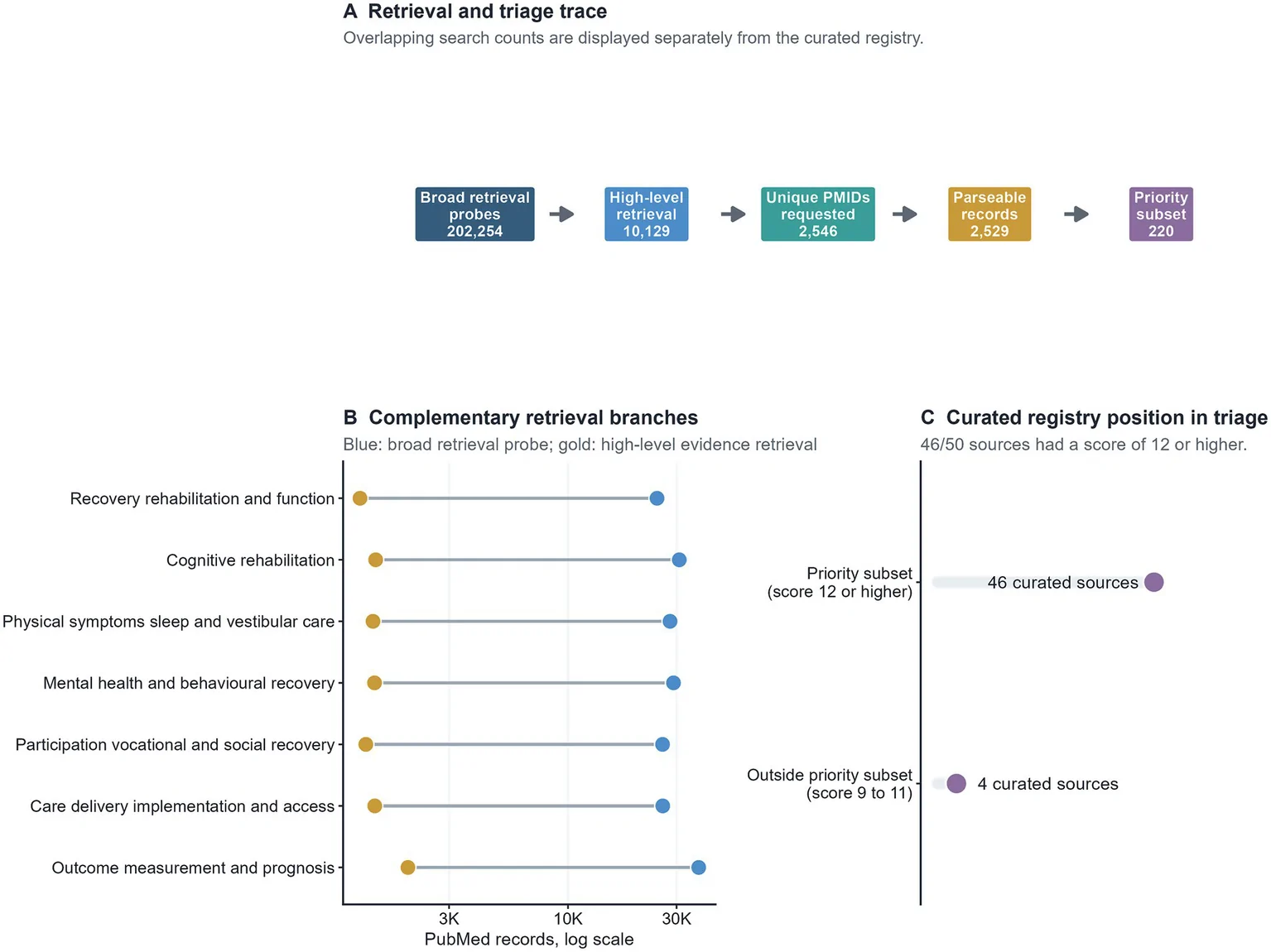
Retrieval traceability and curated-registry position in rule-based triage. **(A)** Retrieval trace from broad probes through high-level retrieval, unique PMIDs, parseable records and the priority subset. **(B)** Complementary broad retrieval probes and high-level evidence retrieval across seven strands. **(C)** Position of illustrative curated-registry sources across priority-score strata.

### Transparent triage

A prespecified priority score combined evidence-type weight (1–5), recency weight (0–3), domain weight (1–2), recovery signal (+2), population signal (+2) and direct-TBI-title signal (+2), with penalties for animal (−10), paediatric-only (−2), acute/surgical (−5) and biomarker-only (−4) signals. The score organized the 2,529 parsed records into a traceable priority subset; component fields and the exact recomputation are reported in Supplementary materials S6, S19. Independent manual relevance validation used prespecified criteria and a stratified sample of 200 records drawn from four priority-score strata (50 records per stratum). Two reviewers independently assessed relevance without reference to the automated priority classification, and discrepancies were resolved by consensus. Concordance review confirmed the original triage assignments and identified no systematic misclassification requiring revision of the principal analyses.

### Curated source registry

Fifty contemporary sources were curated from returned PubMed records to make pre-specified recovery decision contexts inspectable. Eligible candidates were English-language, PubMed-indexed human-TBI guidance, consensus, review or synthesis records with a post-acute recovery, rehabilitation, symptom, participation, outcome or service-delivery signal. Animal/preclinical and non-TBI records and sources restricted to acute resuscitation or neurosurgery were outside the recovery-effect synthesis; one review protocol was retained only as a protocol/methods source and was not interpreted as completed effect evidence. No numerical domain quota was applied. Each source received one title/abstract-audited primary recovery domain, one nominal source role and a source-specific rationale in Supplementary material S15. Registry membership was not defined by dual independent full-text eligibility screening (see Table 2).

**Table 2:** Architecture of the illustrative curated registry by recovery domain and nominal source role.

Recovery domain	Sources, *n*	Guidance	Intervention	Implementation / service	Measurement / prognostic	Protocol / methods
Mental health and behaviour	12	1	5	0	5	1
Symptoms, sleep and physical recovery	9	2	6	0	1	0
Cognitive rehabilitation	7	1	2	0	4	0
Implementation and access	7	1	0	6	0	0
Multidisciplinary rehabilitation	10	5	3	0	2	0
Outcome measurement and prognosis	1	0	0	0	1	0
Participation and social recovery	4	0	0	4	0	0

### Annotation framework

Each curated source was annotated for evidence type, one primary recovery domain, one nominal source role, severity, recovery-time, care-setting and age signals, comorbidity context, treatment stage, surgical/acute-system context, named medication class, fluid-therapy context, neuroprotection/nootropic language, functional outcomes, molecular/biochemical markers, cell types, brain regions and one or more patient-relevant outcome labels. A source could contribute to multiple context or outcome labels but to exactly one primary recovery domain. Source roles were guideline or consensus, intervention synthesis, implementation or service evidence, measurement or prognostic evidence, and protocol or methods source. They were treated as nominal categories and were not aggregated, ranked or interpreted as quality, certainty, net benefit or clinical utility (see Table 3).

**Table 3:** Reporting of age, comorbidity, severity, recovery time and care-setting signals in the illustrative curated registry.

Descriptor	Reported category	Sources, *n*
Age signal	Adult signal	20
Age signal	Mixed adult and paediatric/adolescent signal	5
Age signal	Paediatric or adolescent signal	2
Age signal	Age not stated in title/abstract	23
Comorbidity signal	Explicit comorbidity signal	1
Comorbidity signal	Comorbidity not stated in metadata	49
Injury-severity signal	mTBI or concussion stated	13
Injury-severity signal	Moderate-to-severe TBI stated	7
Injury-severity signal	Severity not stated	30
Recovery-time signal	Subacute or transition stated	3
Recovery-time signal	Chronic or persistent stated	12
Recovery-time signal	Time window not stated	35
Care-setting signal	Rehabilitation or transition setting stated	7
Care-setting signal	Digital or remote setting stated	2
Care-setting signal	Vocational or education setting stated	2
Care-setting signal	Outpatient or community setting stated	1
Care-setting signal	Setting not stated	38

### Protocol governance

Protocol governance and query audit. The recovery framework, seven retrieval strands, operational time boundary, annotation fields and registry-construction rules were specified before final retrieval. A terminology ledger records the operational meanings of TBI, older adolescent, post-acute recovery, clinical decision context, recovery domain, time signal, outcome and setting. The audit trail retains the date, rationale and analytical consequence of every change to a retrieval or annotation rule.

### Data model and missingness

Data model, missingness and overlap. The data model retained a record-level PubMed metadata table, a many-to-many search-strand provenance table, a curated-source annotation table and a long-format outcome-link table. Counts in each panel have an explicit denominator: unique parsed PubMed records for retrieval metrics and 50 curated sources for source-level metrics. Unstated severity, recovery time, care setting, age and comorbidity were represented as reporting categories rather than imputed values. Sources could contribute to more than one outcome category.

### Reproducibility and validation

Retrieval reproducibility was supported by archived queries, PMIDs, XML metadata and deterministic transformations. Bibliographic verification matched the PMID, title, journal, year, publication type and DOI for each curated source. A separate arithmetic implementation check reproduced the stored priority score for all 2,529 parsed records, with no mismatches. Specialists in neurosurgery, neurology, neurorehabilitation and psychiatry independently reviewed all 50 curated sources for source relevance, recovery stage, severity context, treatment history, functional domains and clinical interpretability. Disagreements were resolved by consensus, and the prespecified source-role and primary-domain classifications were confirmed; one review protocol was displayed separately from completed evidence syntheses. These procedures establish source-level human concordance and reproducibility, not prospective clinical validation of treatment effectiveness or patient-level prognosis.

### Visual design and quality assurance

*Figure construction*: Each main figure addresses one prespecified question: retrieval and registry traceability; registry architecture; domain-outcome-setting links; clinical decision-context reporting; nominal source-role architecture; and the translational bridge between pathobiology and recovery research. The six figures use consistent labels and color assignments, while each panel retains an independent analytical purpose.

### Statistical analysis

*Analyses were descriptive*: We calculated retrieval counts, registry composition, clinical decision-context reporting, age and comorbidity signals, treatment-stage and prior-treatment context, functional-marker domains, molecular/biochemical-marker, cell-type and brain-region signals, domain-outcome links, setting signals and nominal source-role distributions. Counts are reported exactly, and no reporting signal was imputed. No treatment effects, certainty ratings, risk-of-bias assessments, causal estimates, or patient-level predictions were calculated.

### Reproducibility and reporting safeguards

Supplementary materials S1–S21 provide the search strategy, retrieval manifest, record-level tables, audited curated registry and source-outcome links, registry-construction and capture audits, expanded metadata-context reporting, figure legends, score-recomputation audit, treatment/marker/cell-context table and validation-status matrix.

## Results

### Retrieval and reconciliation

Retrieval was broad and traceable. The seven broad retrieval probes produced 202,254 overlapping hits, and the high-level retrieval paths produced 10,129 overlapping hits. Deduplication yielded 2,546 unique PMIDs, of which 2,529 returned parseable XML metadata; 17 did not return parseable XML and remain in the missing-record audit. Rule-based triage placed 220 records in the priority subset. Figure 2A separates retrieval, parsing and triage. Figure 2B shows the complementary scale of the seven retrieval strands. Figure 2C shows the position of the curated registry in triage: 46 of 50 sources had a score of 12 or higher and four had scores of 9–11.

### Curated register architecture

The illustrative curated registry consisted of 50 PubMed-indexed sources published between 2020 and 2026. After title/abstract and multidisciplinary re-audit, it contained 10 guidelines or consensus documents, 16 intervention syntheses, 10 implementation or service-evidence sources, 13 measurement or prognostic-evidence sources and one protocol or methods source. Independent clinical review confirmed the prespecified classifications. The PubMed retrieval was not restricted to 5 years; the contemporary registry was selected to represent current recovery decision contexts. Each source-level selection rationale is provided in Supplementary material S15, and no numerical domain quota was used. Figure 3 displays the temporal composition of the retrieved high-level evidence and the architecture of the curated registry.

**Figure 3:**
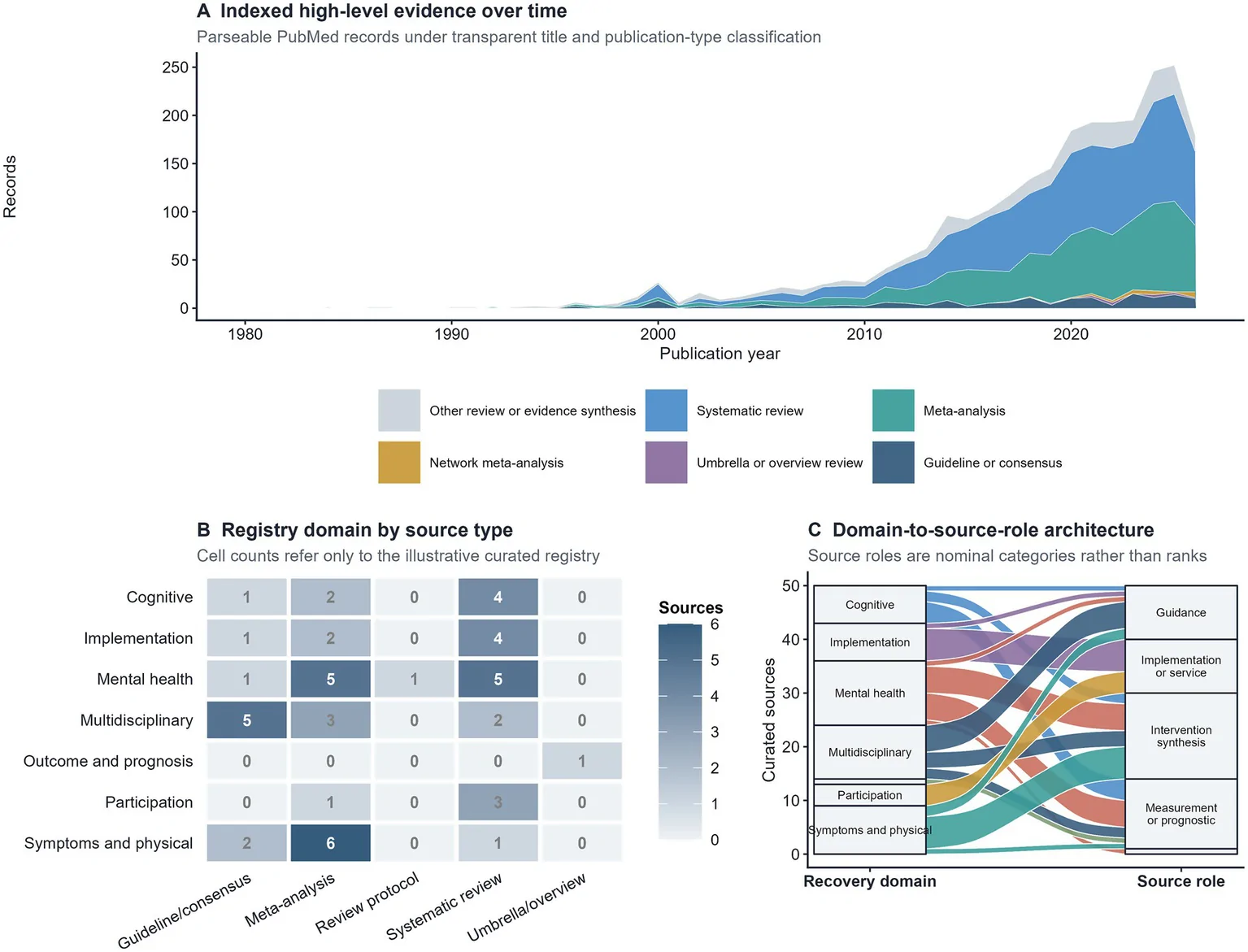
Architecture of the illustrative curated registry. **(A)** Temporal composition of parseable PubMed records by transparent evidence-type classification. **(B)** Source-type counts by recovery domain in the illustrative registry. **(C)** Domain-to-source-role links. Source roles are nominal categories and do not rank certainty, quality or clinical utility.

### Two kinds of incompleteness

The metadata archive distinguishes retrieval status from clinical-context reporting. Seventeen requested PMIDs were not returned as parseable XML on the retrieval date and were retained in the reconciliation audit. Separately, source titles and abstracts frequently did not state severity, recovery time, care setting, age or comorbidity. Figure 2 and Supplementary materials S4–S8 retain both the recovered-record denominator and the field-level reporting pattern.

### Domain coverage

Mental health and behaviour was represented by 12 curated sources, multidisciplinary rehabilitation by 10, symptoms, sleep and physical recovery by nine, cognitive rehabilitation by seven, implementation and access by seven, participation and social recovery by four, and outcome measurement and prognosis by one.

### Outcome and setting maps

The outcome and setting maps show a second level of heterogeneity. Curated sources linked recovery domains with cognition, symptoms or sleep, mental health, participation, function or quality of life, access or implementation, and measurement or stratification labels (Figure 4A). Figure 4B shows that care setting was not stated in 38 of 50 source records; rehabilitation or transition setting was stated in seven, digital or remote setting in two, vocational or education setting in two, and outpatient or community setting in one. Figure 4C shows how outcome labels were represented across nominal source roles.

**Figure 4:**
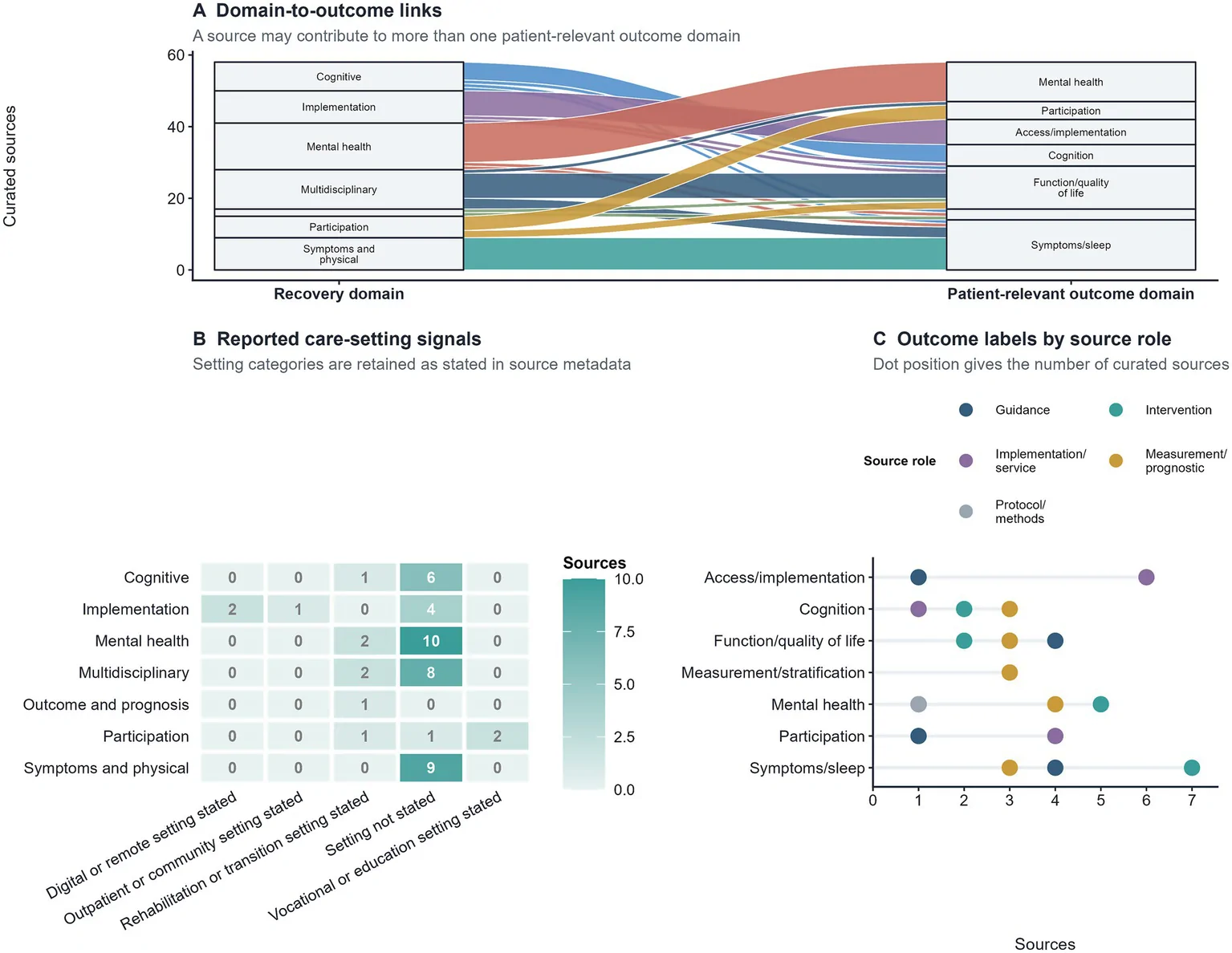
Recovery domains, patient-relevant outcome domains and reported care settings. **(A)** links between recovery domains and patient-relevant outcome domains. **(B)** Reported care-setting signals by recovery domain. **(C)** Outcome labels by source role. A source can contribute to more than one outcome domain.

### Severity and recovery-time reporting

Age language was not stated in 23 sources; 20 had adult language, five had mixed adult/paediatric or adolescent language and two had paediatric or adolescent language. General or pre-existing comorbidity language appeared in one source, while psychiatric, sleep/fatigue/pain and cognitive contexts were retained as separate non-exclusive metadata signals (see Figure 5).

**Figure 5:**
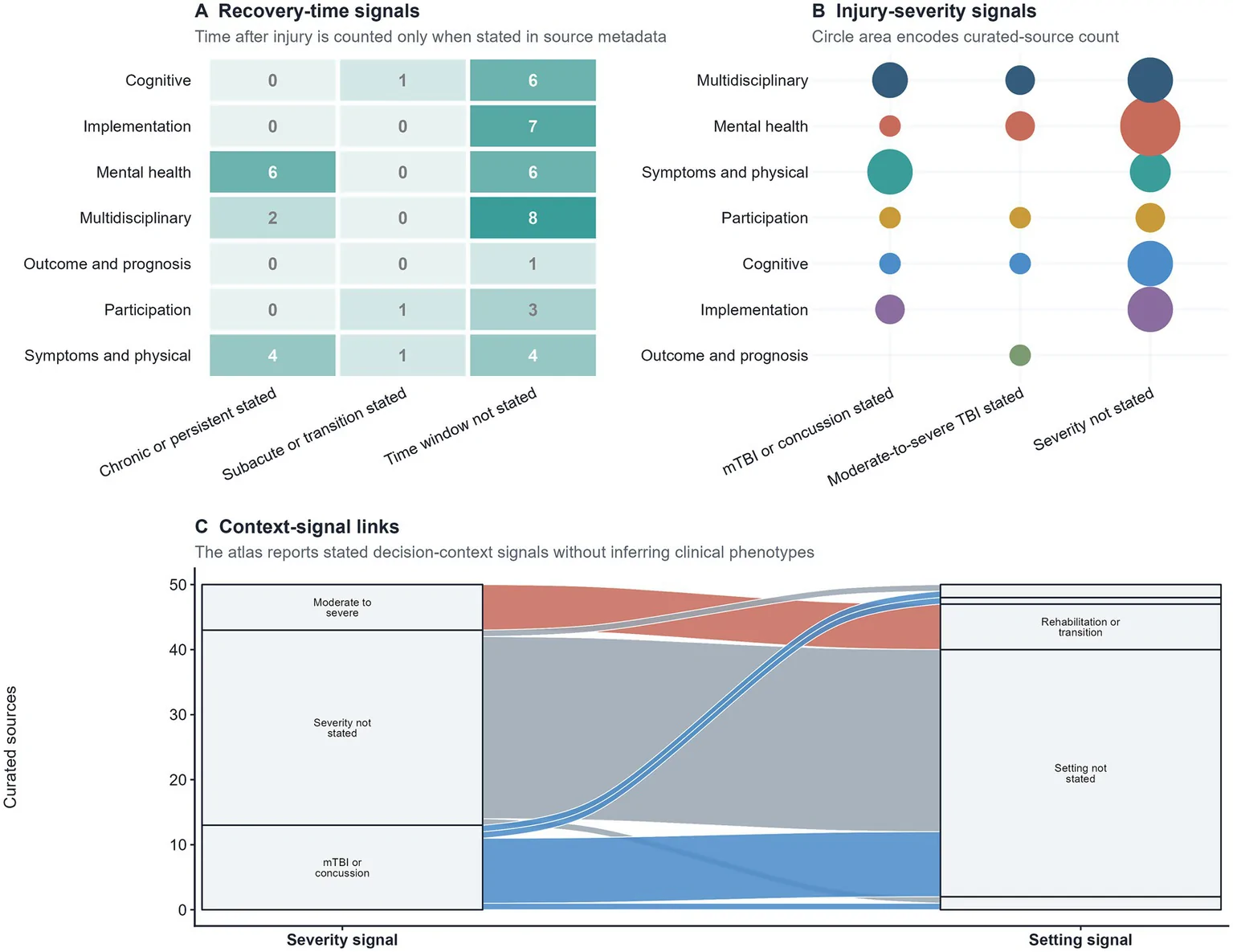
Clinical decision-context signals in curated source metadata. **(A)** Recovery-time signals by recovery domain. **(B)** Injury-severity signals by recovery domain. **(C)** Links between severity and care-setting signals. These descriptors report stated source information and do not define validated biological or clinical phenotypes.

### Inference boundary

Figure 6 displays source-role architecture without rank ordering. Guidance or consensus documents, intervention syntheses, implementation or service evidence, measurement or prognostic evidence, and protocol or methods sources are nominal source categories. Figure 6A links these roles to severity and recovery-time reporting; Figure 6B summarizes their distribution across recovery domains; and Figure 6C links outcome domains to source roles. No source-role score, median or ordinal actionability value is calculated.

**Figure 6:**
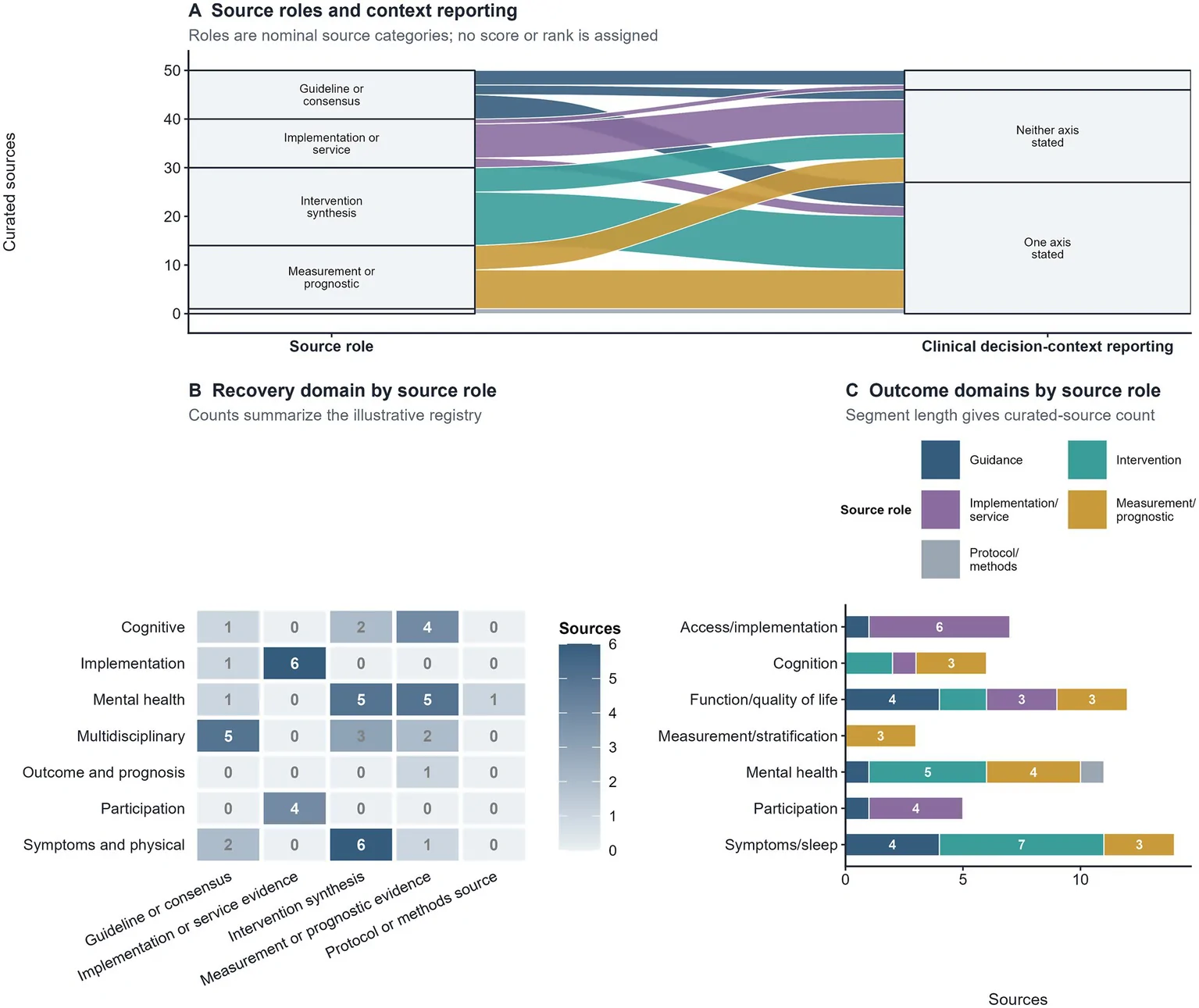
Nominal source-role architecture and clinical decision-context reporting after title/abstract re-audit. **(A)** Five source roles by severity and recovery-time reporting category. **(B)** Recovery domain by source role. **(C)** Patient-relevant outcome domains by source role. No source-role score, median or rank is shown.

### Resistance to rank ordering

Treatment-history context was audited separately from source-role architecture (Supplementary material S20). Surgical or acute-system language appeared in six source records; 12 contained at least one medication-class signal, including methylphenidate, norepinephrine/dopamine reuptake inhibitors, amantadine or general pharmacotherapy. No diuretic/osmotherapy, colloid or crystalloid signal was found in the curated title/abstract metadata. The remaining intervention and measurement sources addressed non-invasive brain stimulation for disorders of consciousness, amantadine, pharmacotherapy for agitation, apathy, cognitive reserve, outcome-measure selection and family-centred sensory stimulation (48–54). Functional outcome domains were represented, but no molecular/biochemical marker, cell-type or brain-region signal was present in the 50 curated records. These zero counts are reporting results, not evidence that the corresponding biology or prior acute therapy is clinically unimportant, and no prior-treatment effect on rehabilitation prognosis was estimated.

### Reproducible seed set

The current registry is traceable at the source level. Every entry has a PMID, DOI, independently reviewed primary recovery domain, nominal source-role category, source-specific selection rationale and reported clinical-context fields. The deterministic score audit reproduced 2,529 of 2,529 record scores exactly, and independent human relevance validation confirmed the original triage assignments across evaluated score strata. The registry-construction audit (Supplementary material S15), registry-capture audit (Supplementary material S16) and validation matrix (Supplementary material S21) make the manual, rule-based and validation stages explicit.

### Human relevance and multidisciplinary review

Independent manual relevance assessment included 200 records sampled across four prespecified priority-score strata (50 records per stratum). Two reviewers independently assessed relevance without access to the automated priority classification. Pre-consensus agreement was 190/200 (95.0%); all 10 disagreements were resolved by consensus. No systematic misclassification requiring revision of the principal analyses was identified. Multidisciplinary review by specialists in neurosurgery, neurology, neurorehabilitation and psychiatry confirmed the prespecified source-role, recovery-domain and clinical-context annotations for all 50 curated sources. These findings validate source-level bibliographic triage and annotation consistency; they do not constitute prospective patient-level clinical validation.

### Implementation and care-transition evidence

Implementation, care-transition and remote-delivery evidence were represented as a distinct source role rather than an extension of intervention effectiveness. The registry includes work on guideline implementation, telehealth, transitional care, post-acute rehabilitation services and telerehabilitation (43–47). These sources address reach, delivery environment, access, participation and service organization. Figure 1 links these questions to the clinical and translational framework.

### Outcome measurement

Outcome measurement warrants a distinct place in post-acute TBI research. Sources in the registry catalogued instruments, prognostic concepts, function, cognition, symptom burden and quality of life. In future studies, outcome definitions, assessment timing, missing-data handling and comparability across recovery domains should be reported as structured fields.

### Consistency across views

Across the six figures, the same source-level pattern persists: indexed high-level literature has expanded, but recovery domains, outcome labels, care settings, age language, comorbidity language and severity-time signals are not represented uniformly. The revised figures distinguish retrieval counts, curated-registry architecture and clinical decision-context reporting, so that visually dense areas are not mistaken for comparative efficacy or certainty.

## Discussion

This work offers a structured account of post-acute TBI recovery evidence: a transparent illustrative registry rather than a single intervention hierarchy. The central contribution is a set of clinically interpretable coordinates for locating source-level evidence across recovery domain, outcome, timing, severity, care setting, age, comorbidity and source role. The registry construction and capture audits make the manual and rule-based stages visible.

The map clarifies why broad statements about TBI rehabilitation can be misleading. Current non-pharmacological and pharmacological approaches address discrete symptoms, cognitive needs, behavioural sequelae, fatigue, sleep, pain, participation and service delivery, but intervention labels alone do not identify the patient group, recovery stage, treatment history, care setting or outcome timing to which a finding applies (5–14). The practical challenge is to align treatment decisions with a documented clinical recovery context.

The most important implication is methodological and clinical. Source-role categories should not be converted into certainty rankings, and source counts should not be converted into claims of treatment benefit. Instead, rehabilitation teams and investigators can use the framework to document severity, time since injury, age, comorbidity, previous acute treatment, functional need, symptom burden, intended treatment dose, delivery setting and patient-relevant outcomes before selecting or evaluating a recovery strategy.

Practical recommendations for clinical research and care are therefore explicit. Rehabilitation assessments should preserve the time since injury, severity language, symptom and functional targets, age group, comorbidity, treatment history and intended care setting. Trials should specify intervention dose, comparator, outcome timing and participation outcomes alongside symptom and cognitive measures. Implementation studies should prespecify reach, adoption, fidelity, equity and resource outcomes for inpatient, outpatient, community, vocational and digital delivery.

The translational framework in Figure 1 links TBI pathobiology to recovery research. Primary mechanical injury initiates metabolic, vascular and immune responses involving microglia, astrocytes, endothelial and neurovascular cells, oligodendroglial lineages and infiltrating myeloid cells; these processes interact with neural repair and persistent recovery symptoms over time (1–3). Figure 1 does not assign cell-type effects from the curated registry. It identifies the mechanistic and clinical fields that must be linked in prospective studies of stratified rehabilitation.

The framework is suited to a programme of translational research. It can structure a full-text extraction form, identify which recovery coordinates are absent from published reports, guide eligibility and endpoint selection for rehabilitation trials, and align implementation studies with the clinical pathway in which an intervention will be used. The same intervention may yield different results when recovery stage, symptom profile, care setting and patient priorities differ.

This study has limitations. The search used PubMed and English-language records, and the 50-source registry was curated rather than derived from a completed multi-database, dual-reviewer full-text systematic review. Although the rule-based triage underwent independent human relevance validation and the curated annotations underwent multidisciplinary clinical review, both validation steps were source-level rather than patient-level. Metadata reporting cannot establish risk of bias, overlap between primary studies, treatment-effect direction, effects of prior surgery or medication, outcome harmonization, certainty of evidence or net benefit. Molecular/biochemical markers, cell types and brain regions were absent from the curated title/abstract metadata and are not atlas-derived results. The study did not include patient-level data or prospective clinical validation; such validation remains necessary before any use as a clinical decision-support or prognostic system.

These limitations define the appropriate use of the revised resource. TBI-RECOVER is intended to support precise evidence-review questions, transparent source selection and prospective clinical and translational study design. It does not provide a clinical decision algorithm, a treatment recommendation or a patient-level prognosis estimate.

The principal strengths are traceability and interpretability. The query history, PMID map, retrieved metadata, registry-construction audit, context-reporting table, revised figures and source-data files preserve the provenance of every displayed count. The revised title, objectives, tables and figures align the manuscript with this source-level design.

Clinical and translational applications follow directly from the revised framework: record the clinical recovery context, distinguish source role from evidence certainty, preserve treatment history as a contextual variable, and use prospective cohorts or trials to test whether biological and clinical profiles modify rehabilitation response. These principles can improve communication among neurosurgeons, neurologists, rehabilitation specialists, psychiatrists and implementation teams without presenting the registry as a validated decision-support system.

## Conclusion

TBI-RECOVER is a post-acute TBI recovery evidence registry that describes how contemporary high-level sources are distributed across clinical decision-context signals, recovery domains, outcomes, care settings and nominal source roles. The PubMed search spans database inception, and the illustrative 50-source registry is traceable through registry-construction and capture audits, exact score recomputation, independent human relevance validation and multidisciplinary clinical review.

Its immediate contribution is to define the fields required for rigorous post-acute TBI research: recovery stage, severity, age, comorbidity, prior treatment, functional and symptom profile, delivery setting, treatment dose and patient-relevant outcomes. These fields provide a practical bridge from mechanistic research to stratified rehabilitation and implementation studies.

## Data Availability

The original contributions presented in the study are included in the article/Supplementary material, further inquiries can be directed to the corresponding authors.
